# Comprehensive non-contrast MR assessment of the iliofemoral arteries with visualization of vascular calcifications in TAVR candidates - initial experience

**DOI:** 10.1186/1532-429X-18-S1-P358

**Published:** 2016-01-27

**Authors:** Marcos P Ferreira Botelho, Jeremy D Collins, Robert R Edelman, Ioannis Koktzoglou, Ian G Murphy, James C Carr

**Affiliations:** 1 Radiology, Northwestern University, Chicago, IL USA; 2 Radiology, NorthShore University HealthSystem, Evanston, IL USA

## Background

Transcatheter aortic valve replacement (TAVR) has emerged as an accepted therapeutic option for moderate and high-risk patients with severe aortic stenosis. In addition to vessel caliber, the presence and configuration of vascular calcifications by the iliofemoral access site is part of routine pre-interventional assessment with CTA and catheter angiography. Non-contrast MR angiography has been proven useful for evaluation of lower extremity peripheral vascular disease, however it cannot identify vascular calcifications using currently available sequences. Neutral contrast 3D and 3D point-wise encoding time reduction (PETRA) are novel MR techniques that can detect vascular calcifications and enable projection-like displays for review, similar to CT. We hypothesized that these techniques, associated with non-contrast Quiescent Interval Slice-Selective (QISS) MRA may be useful alternatives for pre-surgical planning in patients considered for TAVR.

## Methods

We prospectively enrolled 5 patients considered for TAVR for this pilot study (mean age 75 years, 3 males). MR imaging was acquired in a 1.5T magnet. Non-contrast MRA was performed using a prototype ECG gated QISS sequence, optimized for the pelvic and femoral vasculature with 3 mm slices, Cartesian sampling. Stable versions of PETRA with 1 mm axial slices and neutral contrast 3D MRI with 0.8 mm coronal slices were acquired. Scan times were noted. A single reader compared MR images to CTA in terms of calcification visualization, patency and severity of segments of stenosis, using minimal orthogonal measurements.

## Results

All MR techniques were feasible in all patients. Total MR scan times ranged from 13 to 17 minutes. There was comparable qualitative visualization of the calcifications among CTA, PETRA and the neutral contrast technique, including segments with circumferential calcification. Neutral contrast demonstrated less artifacts from superficial soft tissues compared to PETRA. Severe or complete segmental stenoses were present in 2 of the patients, detected both by CTA and QISS. Orthogonal luminal measurement differences between CTA and QISS ranged from none (42% of all measurements) to 2 mm (12%).

## Conclusions

Calcifications can be adequately evaluated in presurgical planning for TAVR using both Neutral Contrast and PETRA, with similar visualization compared with CTA. QISS MRA and CTA have comparable accuracy detecting stenosis of the pelvic vasculature of these patients. These non-contrast MR techniques have great potential as alternatives for assessment of vascular disease in TAVR candidates. Further technical development is ongoing for calcification MRI techniques.

**Figure 1:**
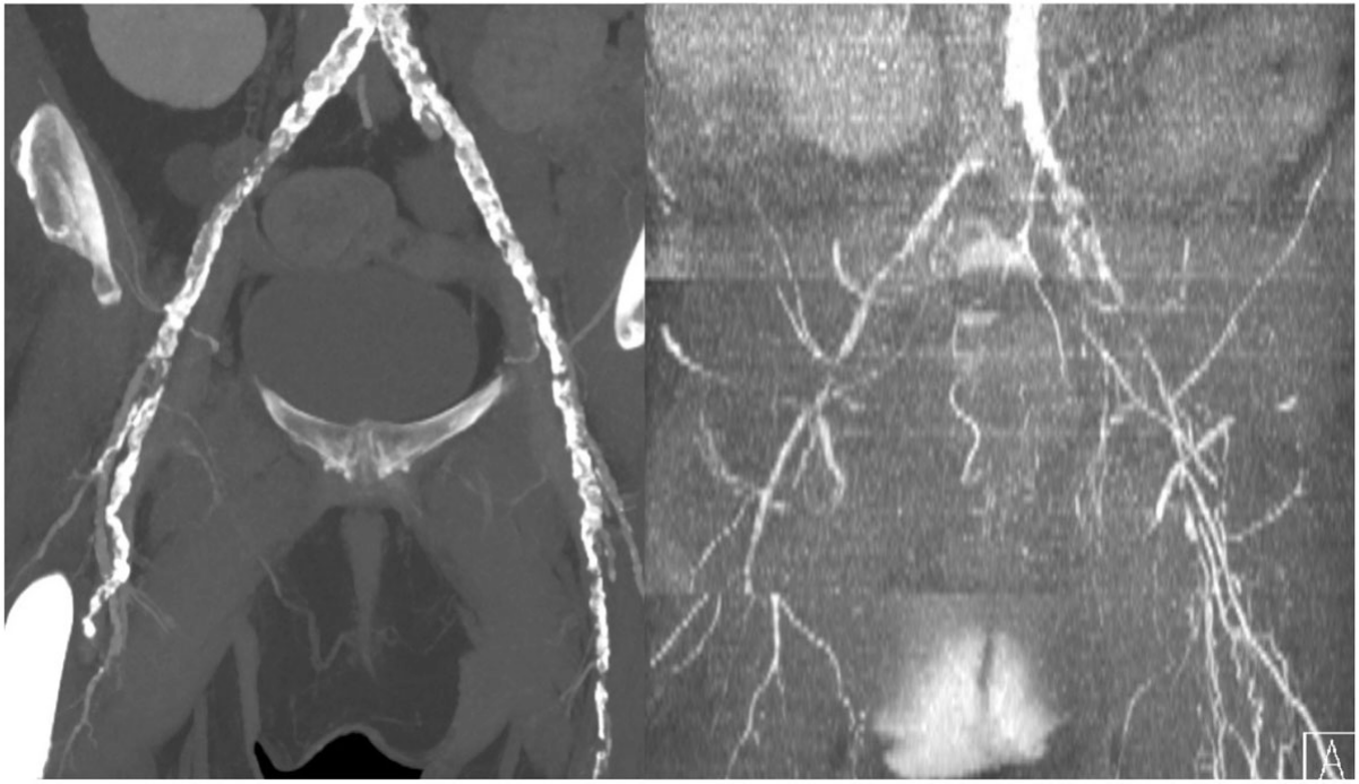
**MIP CTA and MIP QISS MRA in a 54 yo female**. CTA demonstrates the arterial system and calcifications, but extensive atherosclerosis limits evaluation of the vessel lumen. Absent flow in the proximal right common iliac artery is well identified by QISS MRA.

**Figure 2:**
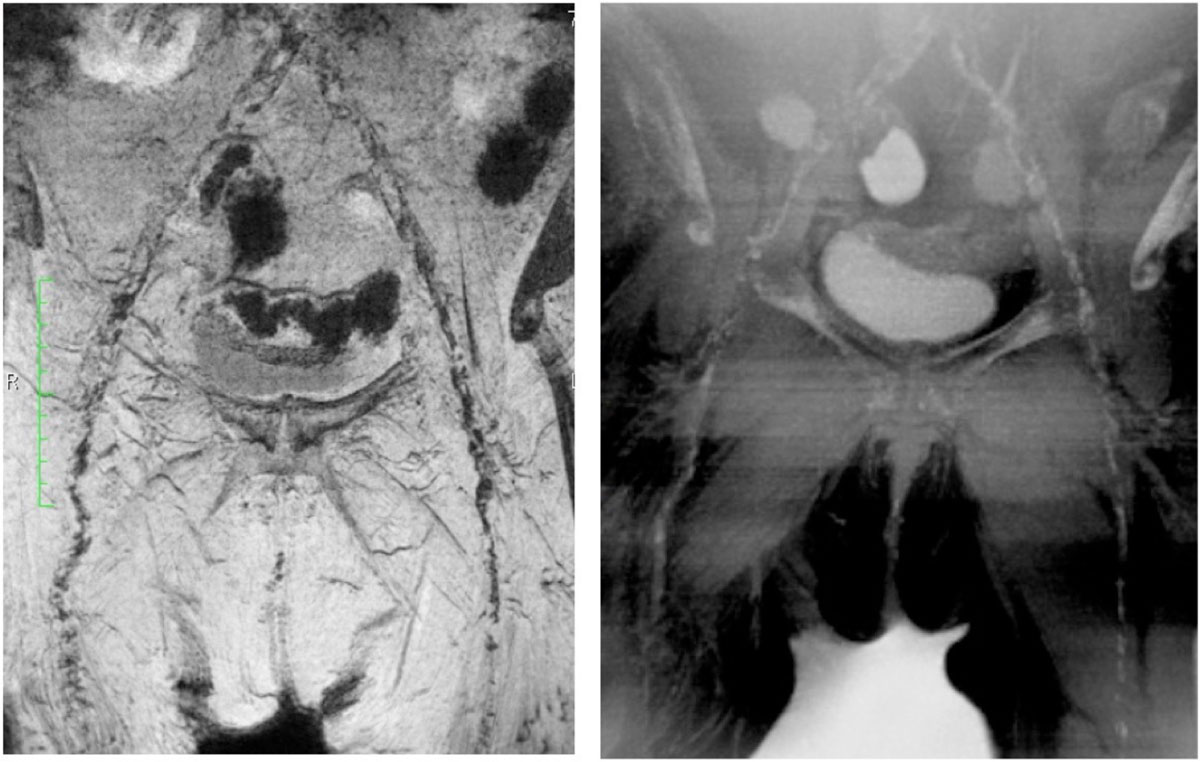
**MinIP Neutral Contrast and inverted B/W MinIP PETRA in the same patient**. Extensive arterial calcification is clearly demonstrated in a similar manner, but with better contrast and less artifact with the first technique.

